# Association Between Solid Organ Transplantation and Oral Candidiasis: A Systematic Review and Meta‐Analysis

**DOI:** 10.1111/scd.70157

**Published:** 2026-03-23

**Authors:** Neha Shah, Sangeeta Sinha, Abhay Sonthalia, Divesh Sardana

**Affiliations:** ^1^ Department of Oral Pathology Guru Nanak Institute of Dental Sciences and Research (GNIDSR) Kolkata West Bengal India; ^2^ Private Practitioner Kolkata West Bengal India; ^3^ Department of Pediatric Dentistry Indiana University School of Dentistry and James Whitcomb Riley Hospital for Children Indianapolis Indiana USA

**Keywords:** antifungal agents, Candida, fungi, immunocompromised host, immunosuppression therapy, oral candidiasis, transplantation

## Abstract

**Aim:**

To summarize the evidence on the association of oral candidiasis with solid organ transplantation in case‐control and cohort studies.

**Methods:**

The quality of studies was assessed using the Newcastle–Ottawa scale. Random‐effects restricted maximum likelihood model or Fixed‐effects Mantel–Haenszel models were used to pool the results appropriately, and sensitivity analysis, subgroup analysis, and publication bias were assessed. The certainty of evidence was assessed using the GRADE approach.

**Results:**

The pooled OR of candidiasis in immunosuppressed individuals receiving solid organ transplants compared to controls was found to be 3.52 (95% CI: 1.92, 6.46; *p* < 0.05). The pooled OR of oral candidiasis in kidney transplant recipients compared to controls was 3.53 (95% CI: 1.92, 6.49; *p* < 0.05); for liver, 20.12 (95% CI: 3.83, 105.74; *p* < 0 .05), and heart, 152.01 (95% CI: 17.71, 1304.96; *p* < 0.05).

**Conclusions:**

There was moderate certainty of evidence that there was an increased risk of oral candidiasis in immunosuppressed patients who received solid organ transplants compared to healthy controls. Similarly, the evidence was moderate for subgroup analysis regarding the kidney transplant recipients, liver transplant recipients, and heart transplant recipients. Due to the lack of current research, future research should evaluate the risk of oral candidiasis in patients receiving pancreas or small bowel transplants. Also, future studies should evaluate the role of prophylactic antifungals in immunosuppressed solid organ transplant recipients.

**Trial Registration:**

PROSPERO number: CRD42022363816.

## 1 Introduction

With the evolution in transplant medicine, the rate of solid organ transplantation (kidney, heart, lung, liver, pancreas, and small bowel) has increased from 6.82 individuals per million in 2000 to 28.87 individuals per million in 2024, with absolute numbers increasing from 41,259 in 2000 to 173,848 in 2024 [1]. The actual figures might be significantly higher, given that data from some countries were not included in the repository. Immunosuppression induction (*immediately after organ transplant to prevent early rejection*) and immunosuppression maintenance (*after the induction phase, continues for life, and prevents acute and chronic rejection*) are often the cornerstone of a successful organ transplantation and thus reduce mortality and morbidity in patients with organ transplantation [2, 3]. Consequent to immunosuppression, solid organ transplant recipients experience a high prevalence and incidence of infections during the immediate first year post‐transplantation. These infections might be bacterial (62.6%), viral (29.5%), fungal (7.5%), and parasitic (0.5%) as reported in a cohort study on a sample size of 2761 organ recipients [4]. In a large multicentric longitudinal study, it was found that invasive candidiasis was the most common fungal infection (53%) in solid organ transplant recipients, followed in order by invasive aspergillosis (19%), cryptococcosis (8%), non‐*Aspergillus* molds (8%), endemic fungus (5%), and zygomycosis (2%) [5]. Candida infection might present as a spectrum of less serious mucocutaneous infections to life‐threatening conditions like candidemia (presence of fungus in the bloodstream) or invasive candidiasis of the deeper tissues and organs, depending on the host's immunity and cellular defense [6, 7].

Candida is a commensal fungus present in the oral cavity, skin, gastrointestinal tract, and vagina, and comprises nearly 200 species of which roughly 10% are pathogenic to humans [7]. More than 90% of these fungal infections are known to be caused by only five Candida species: *Candida albicans*, *Candida glabrata*, *Candida krusei, Candida parapsilosis, and Candida tropicalis* [8, 9]. The oral colonization of Candida has been reported to be higher, with 39% of the individuals being carriers, of which the majority might be colonized by *Candida albicans* (89%), followed by *Candida guilliermondi* (5%), *Candida lusitaniae* (3%), and *Candida parapsilosis* (3%) [10]. There are other non‐albicans *Candida* species that have been isolated from the oral cavity in patients with oral candidiasis, which are: *Candida glabrata, Candida tropicalis, Candida kefyr, Candida dubliniensis, Candida krusei*, to name a few of them [11]. In patients with healthy immune response, *Candida* lives as a commensal with other organisms and is unable to cause any pathology due to the host immune response; however, in events of immune stress and compromised host defenses (hormonal disorders like diabetes, geriatric patients, steroid therapy, etc.), *Candida* might become pathogenic and cause oral candidiasis [12].

Organ transplantation (solid or hematopoietic) is one such condition where the host's immunity is compromised due to the immunosuppressant drugs used for induction and maintenance to prevent the rejection of the graft. While some reviews have assessed the risk and burden of systemic fungal infections or colonization in solid organ transplant patients [13, 14, 15, 16], none of them, to the best of our knowledge, have focused on the association of oral candidiasis with solid organ transplantation or evaluated the association between oral candidiasis and the type of solid organ transplanted. The purpose of the present systematic review and meta‐analysis was to summarize the evidence on the association of oral candidiasis with solid organ transplantation in case‐control and cohort studies.

## 2 Materials and Methods

### 2.1 Protocol and Registration

The methodology of the current review was formulated in advance by adhering to the Cochrane Handbook and documented in the protocol [17]. Subsequently, the protocol was designed a priori and registered at the International Prospective Register of Systematic Reviews. The review is being reported as per the PRISMA (Preferred Reporting Items of Systematic Reviews and Meta‐analyses) statement and checklist [18].

### 2.2 Study Eligibility Criteria

The PECOS schema was used to describe the inclusion of eligible studies as follows:

- P (Participants/Population): Patients who received solid organ transplants.
- E (Exposure): Solid organ transplantation (Heart, Lung, Liver, Kidney, Small Bowel, Pancreas).
- C (Comparison): Patients without solid organ transplant (for case‐control studies) and patients before solid organ transplantation (for longitudinal studies).
- (Outcome): The risk of oral candidiasis in the exposed versus the control group (for case‐control studies) and the risk of candidiasis before and after organ transplantation (for longitudinal studies) were considered as the primary outcome. Since there can be other oral mucocutaneous disorders having similar signs and symptoms as oral candidiasis, only studies that diagnosed candidiasis based on both clinical criteria and confirmed using mycological examination or biopsy, or response to antifungal treatments were included.
- S (Study Design): Only case‐control and cohort studies were included in the present review. Due to the nature of the exposure (solid organ transplantation), we did not anticipate any randomized clinical trials and were thus planned not to be included.

Exclusion criteria: in vitro studies, case reports, case series, narrative or systematic reviews, and studies done on hematopoietic stem cell transplants.

### 2.3 Information Sources and Literature Search

A systematic search of the literature was performed on five electronic databases using broad MeSH terms and keywords on March 7, 2024, by two investigators (S.D. and S.N.). The five databases searched were: Medline (via Ovid), Embase (via Ovid), Scopus, PubMed, and Web of Science. The reference lists of the included articles were also checked to identify any potential articles that could have been missed in the electronic search. To identify grey literature, www.opengrey.eu, and Google Scholar were also searched for any unpublished material with broad keywords including thesis, dissertations, and conference abstracts. The search strategy for all the databases is presented in Appendix S1.

### 2.4 Study Selection

EndNote X 9.2 software for Windows (Clarivate Analytics, Philadelphia, PA, USA) was used to import the results obtained through the searching of electronic databases, journals, and gray literature. After the removal of duplicates, the records were scanned by titles and abstracts by two independent investigators (S.N. and S.S.) to determine the eligibility for full text reading. If the reading of the abstract provided an unequivocal interpretation regarding inclusion or exclusion, the record was subjected to full‐text reading. A third investigator (S.D.) was consulted in case of any discrepancy between the two investigators. Cohen's kappa coefficient (κ) was calculated to establish the level of interrater agreement at full‐text reading stage [19].

### 2.5 Data Items

Information and data pertaining to the following parameters were extracted from each included study: author, year of study, country of study, type of study, sample size [total, study group, control], type of organ transplant, mean age and/or range, gender distribution, diagnostic method for Candidiasis, type of oral candidiasis, site of intra‐oral involvement, species of Candida, immunosuppressive drug regime, incidence/prevalence in the cases/controls, follow‐up periods, comorbidities/confounders in exposed and non‐exposed groups.

### 2.6 Risk of Bias in Individual Studies

The quality of the included studies was assessed using the Newcastle Ottawa Scale (NOS) [20] and assigned stars for each parameter under three domains, wherever applicable, by two authors independently (S.N. and S.S.). Any discrepancies regarding the assessments were mutually discussed, and consensus was achieved through opinion from the third investigator (S.D.). The included articles were evaluated under the categories of selection (maximum of 4 stars), comparability (maximum of 2 stars), and exposure (maximum of 3 stars). The stars were assigned if the criteria for the particular domain were met. For case‐control studies, the study was awarded one * (star) each if the definition of the cases was adequate (solid organ transplant recipients diagnosed with oral candidiasis through clinical examination and confirmed by swab/culture/smear/biopsy/other advanced diagnostic methods), cases were representative (representativeness of the cases was defined as selective cases that represented the general population), definition of controls were adequate (control was defined as healthy individuals without history of candidiasis prior to enrolment in the study) and selected controls were representative of the general pool of the healthy population. If the study strictly controlled for the most important factor or did an analysis to control for the factor (defined in our review as “Diabetes, smoking, removable prosthesis or any other immunosuppressed conditions” that could also cause the outcome viz. Oral Candidiasis) it was awarded one star, if the study considered all three factors and if the study controlled for additional factors that could predispose an individual to oral candidiasis (like age and gender), it was awarded an additional star if it accounted for both age and gender in the domain of comparability. The study was awarded a star each if there was a robust ascertainment of the exposure in cases and controls using the same criteria based on surgical records and structured interviews of cases/controls. The parameter of non‐response rate under the category of exposure was not rated as it was not applicable to the review.

### 2.7 Summary Measures and Methods of Analysis

The primary outcomes of this review were either binary or continuous and were expressed as Odds Ratio (OR), respectively, with their corresponding 95% Confidence Intervals (CI). Adequately homogeneous and similar outcomes in the included studies were analyzed quantitatively by pooling the results. Aggregate data extracted from the studies were used to perform the quantitative synthesis. Two authors (S.N. and S.S.) independently extracted the data on a piloted proforma, and any discrepancies were sorted out by mutual discussion or consultation with the third author if required. Statistical heterogeneity of the included studies was evaluated using the χ^2^‐based Q‐statistic method and I^2^ measurement, with significance indicated by an alpha of 0.05. The planned approach for quantitative synthesis was the DerSimonian and Laird random‐effects model using the restricted maximum likelihood (REML) approach if the number of studies was more than 3, or the Fixed‐effects Mantel–Haenzel model if the number of studies was less than 3.

### 2.8 Certainty of Evidence

The GRADE approach [21] was used to generate the synthesis of evidence based on the assessment in the following domains: Study design, risk of bias, inconsistency, indirectness, imprecision, and other factors like the strength of association. Since the studies included in the meta‐analysis were only case‐control designs, an initial assessment of “*low*” was provided and further downgraded or upgraded depending on the other domains.

## 3 Results

### 3.1 Study Selection

**FIGURE 1:**
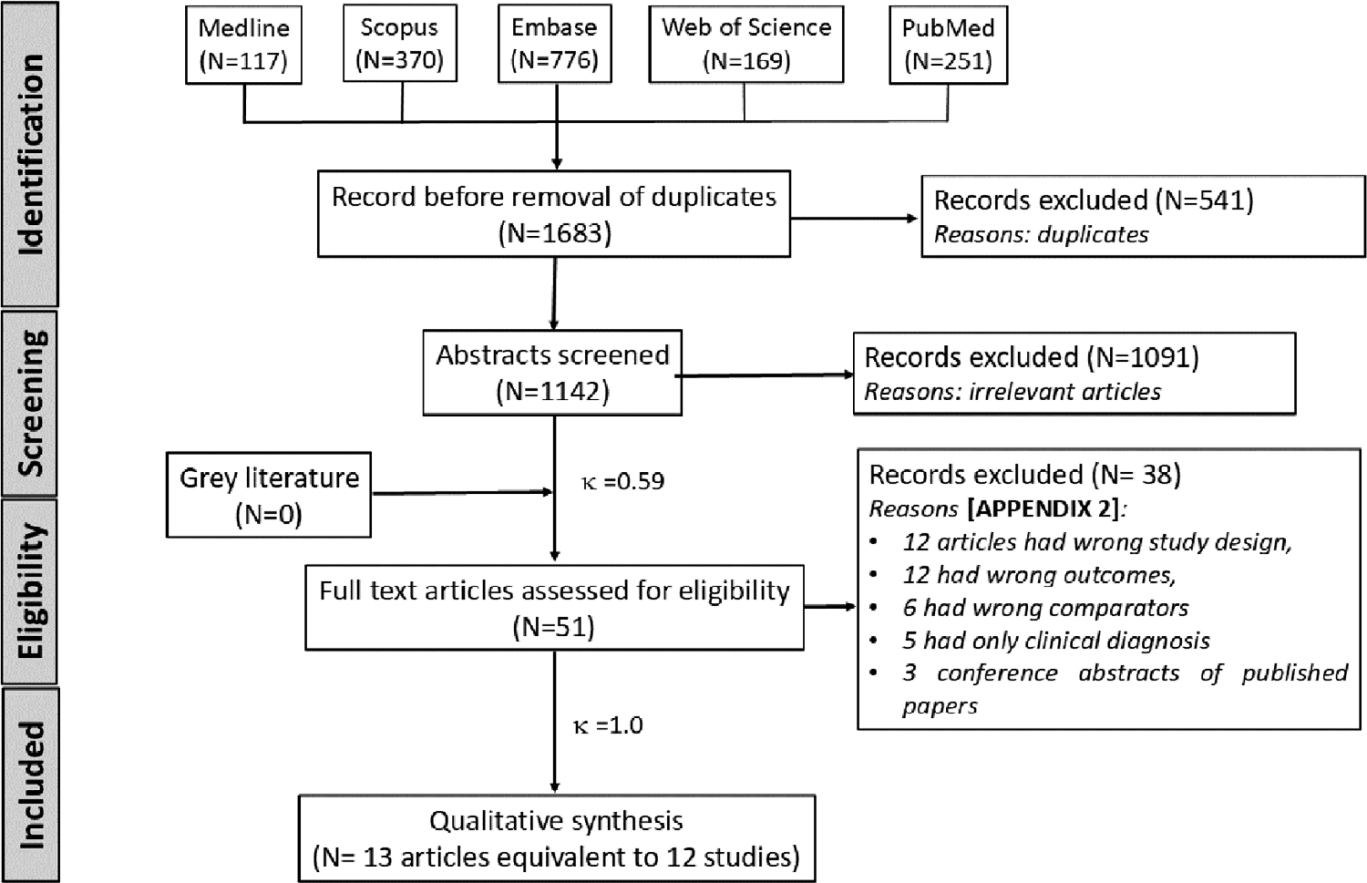
PRISMA flow diagram of the study selection process

The article selection process is provided in the flow summary (Figure 1). A total of 1683 articles were identified after the initial electronic database search, out of which 1142 studies were screened for titles and abstracts after the removal of duplicates. Among these, 51 potentially eligible studies were subjected to full‐text reading, but finally, only 13 articles (equal to 12 studies) met our selection criteria and were included in the present review [22, 23, 24, 25, 26, 27, 28, 29, 30, 31, 32, 33, 34]. One of the included studies strongly appeared to be salami sliced into two different articles [29, 30], but the results and data of the study were considered once in the review to avoid duplication and inflation of the weight of the study in the synthesis of the results. The Cohen's kappa (κ) value was determined to be 1.00 after full‐text reading, indicating a perfect level of agreement. The reasons behind the exclusion of the studies are described in Appendix S2.

### 3.2 Characteristics of the Included Studies

The selected studies were carried out in Austria, England, Iran, Poland, Saudi Arabia, Spain, Turkey, and the United States. Table 1 provides a descriptive summary of the included studies in the current review. Among the 12 included studies, nine were case‐control studies that compared the risk of oral candidiasis between individuals undergoing solid organ transplant and individuals without a transplant, [22, 23, 24, 25, 26, 27, 28, 29, 30, 31] whereas three were cohort studies, [32, 33, 34] where the risk of oral candidiasis was assessed before and after solid organ transplant. The number of patients who received solid organ transplants was 1997. The most commonly transplanted organ was the kidney (12 studies; *n* = 1618), [22, 23, 24, 25, 26, 27, 28, 29, 30, 31, 32, 33, 34] followed by the liver (3 studies; *n* = 152), [25, 28, 33] heart (3 studies; *n* = 135), [25, 26, 33] and lung (1 study; *n* = 92) [33].One of the included studies [33] provided data on solid organ transplants along with bone marrow transplants, of which data from 81 solid organ transplant recipients were extracted and included in the present review.

**TABLE 1:** Characteristics of included studies.

Author Year, Country	Type of study	Sample size (*N*)	Type of organ transplant	Mean age (years) unless indicated	Gender distribution	Diagnostic method for detection of candidiasis	Candida species isolated	Immunosuppressive drug regimen	Incidence/ Prevalence of oral candidiasis	Type of oral candidiasis	Follow‐up period/ post‐transplant period	Significant comorbidities/ Confounders in transplant and control groups
King et al. (1994) [22] England	Case‐control	Transplant group = 159 Control group = 160	Kidney	Transplant group = 48.2 (SD: 13.3) Control group = 41.4 (SD: 14.7)	Transplant group: Males = 104 Females = 55 Control group: Males = 101 Females = 59	Clinical examination, smear examination, culture on Sabouraud's medium and response to antifungal therapy	Not specified	Azathioprine and Prednisolone combination (151 patients) Only Cyclosporine A (8 patients)	Prevalence: Transplant group = 12.6% (*n* = 20) Control group = 2.5% (*n* = 4)	Erythematous candidiasis: Transplant group = 3.8% (*n* = 6); Control group = 0% Denture‐induced stomatitis: Transplant group = 3.8% (*n* = 6); Control group = 1.3% (*n* = 2) Pseudomembranous candidiasis: Transplant group = 1.9% (*n* = 3); Control group = 0.6% (*n *= 1)	68.3 (SD: 53) months	Active smoking: Transplant group = 14.5%; Control group = 37.5% Smoking history: Transplant group = 45.3%); Control group = 58.1% Alcohol (units/week): Transplant group = 3.2 (SD: 5.6); Control group: 5.6 (SD:11.7)
Gülec et al. (2003) [23] Turkey	Case‐control	Transplant group = 102 Control group = 88	Kidney	Transplant group = 31.9 (SD: 10.3) Control group = 32.5 (SD: 11.2)	Transplant group: Males = 68 Females = 34 Control group: Males = 58 Females = 30	Clinical examination, smear examination, culture on Sabouraud's medium, Germ tube test	*C. albicans* *C. parapsilosis* *C. krusei* *C. tropicalis*	Combinations or alone: Azathioprine Cyclosporine Mycophenolate mofetil Prednisolone Tacrolimus	Prevalence: Transplant group = 25.5% (*n* = 26) Control group = 12.5% (*n* = 11)	No subgroup analysis mentioned	53.6 (SD: 54.6) months	Not specified/ no analysis presented
Al‐Mohaya et al. (2009) [24] Saudi Arabia	Case‐control	Transplant group = 58 Control group = 52	Kidney	Transplant group = 39.2 (SD: 12.8) Control group = 37.1 (SD: 11.6)	Transplant group: Males = 36 Females = 22 Control group: Males = 34 Females = 18	Clinical examination, mucosal swab and smear examination, culture on Sabouraud's medium, Germ tube test, species identification by commercially available yeats identification system API 20C AUX 21 (bioMerieux, Marcyl, Etiole, France)	*C. albicans* *C. dubliniesis* *C. famata*	Prednisolone (58 patients) Also, Cyclosporine (56 patients)	Prevalence: Transplant group = 17.2% (*n* = 10) Control group = 0% (*n* = 0)	Erythematous candidiasis: Transplant group = 15.5% (*n* = 9); Control group = 0% (*n* = 0) Candida associated Angular cheilitis: Transplant group = 1.7% (*n* = 1); Control group = 0% (*n* = 0)	51.6 (SD: 31.9) months	Smoking balanced across groups.
Antoniewicz et al. (2009) [25] Austria	Case‐control	Transplant group = 400 Control group = 405	Kidney (*n* = 151) Heart (*n* = 116) Lung (*n* = 91) Liver (*n* = 42)	Transplant group = 56 (SD: not mentioned) Kidney transplant group = 55.9 Heart transplant group = 58.2 Lung transplant group = 52.1 Liver transplant group = 58.4 Control group = 56.7	Transplant group: Males = 256 Females = 144 Control group: Males = 259 Females = 146	Clinical examination, mucosal swab and smear examination, culture on Sabouraud's medium, species identification by API system (bioMerieux, Marcyl, Etiole, France). Data from symptomatic and smear positive or microbiological positive is presented.	*C. albicans* *C. glabrata* *C. parapsilosis* *C. tropicalis* *C. krusei* *C. lusitaniae* *C. dubliniensis*	Combined immunosuppressive regime, usually a triple combination therapy containing corticosteroids plus a calcineurin inhibitor, i.e., cyclosporine (CSA) or tacrolimus (TAC), plus an antiproliferative drug, i.e., mycophenolatmofetil (MMF) or azathioprin (Aza), or a regimen containing an mTOR antagonist	Prevalence: Transplant group = 21.5% (*n* = 86)^b^ Control group = 0% (*n* = 0) Kidney transplant group = 21.9% (*n* = 33) Heart transplant group = 21.6% (*n* = 25) Lung transplant group = 23.1% (*n* = 21) Liver transplant group = 16.7% (*n* = 7)	Pseudomembranous candidiasis: Transplant group = 14% Erythematous candidiasis: Transplant group = 4% Hyperplastic candidiasis: Transplant group = 3% Angular cheilitis: Transplant group = 8% Control group: 0% for all subtypes	5.2 years (SD: not mentioned)	Not specified/ no analysis presented
Dongari‐Bagtzoglou et al. (2009) [26] USA	Case‐control	Transplant group = 90 Control group = 72	Kidney (*n* = 81) Heart (*n* = 9)	Kidney transplant group = 52.7 (SD: 11.9) Heart transplant group = 56.4 (SD: 12.3) Control group = 53 (SD: 10)	Kidney Transplant group: Males = 47 Females = 34 Heart transplant group: Males = 5 Females = 4 Control group: Males = 29 Females = 43	Clinical examination, mucosal swab, culture on Chromagar, germ tube test, bio‐typing, polymerase chain reaction assays and standard sugar assimilation assay profiles (RapID Yeast Plus System, Lenexa, KS)	*C. albicans* *C. glabrata* C*. lambica* *C. rugosa* *C. krusei* *C. tropicalis*	Combinations or alone: Azathioprine Cyclosporine Mycophenolate Prednisolone Sirolimus Tacrolimus	Prevalence: Transplant group = 7.8% (*n* = 7) Control group = 0% (*n* = 0) Kidney transplant group = 7.4% (*n* = 6) Heart transplant group = 11.1% (*n* = 1)	Erythematous candidiasis	Kidney transplant group = 6.8 (SD: 4.8) years Heart transplant group = 8.1 (SD: 5.3) years	Diabetes: Kidney transplant group = 54% Heart transplant group = 67% Control group = 3% Former smokers: Kidney transplant group = 41% Heart transplant group = 75% Control group = 0%
Gülec et al. (2010) [27] Turkey	Case‐control	Transplant group = 100 Control group = 79	Kidney	Transplant group = 35 (SD: 11) Control group = 34 (SD: 10)	Transplant group: Males = 79 Females = 21 Control group: Males = 56 Females = 23	Clinical examination along with KOH staining, smear examination, culture, biopsy	*C. albicans*	Combinations or alone: Azathioprine Cyclosporine Mycophenolate Prednisolone Sirolimus Tacrolimus	Prevalence: Transplant group = 26% (*n* = 26) Control group = 8.8% (*n* = 7)	Unclear distribution between the transplant group and control group	59.7 (SD: 56.9) months	Diabetes: Transplant group = 10% Control group = 0% Smoking: Transplant group = 20% Control group = 43% Alcohol consumption: not significant
Olczak‐Kowalczyk et al. (2010)^a^ [28] Poland	Case‐ control	Transplant group = 185 Control group = 70	Kidney (*n* = 105) Liver (*n* = 80)	Transplant group = 13.1 (SD: 4.2) Control group = 10.8 (SD: 4.2)	Not specified	Clinical examination, mucosal swabs, culture on Sabouraud's medium, species identification by ID 32C test (bioMerieux).	*C. albicans* *C. parapsilosis* *C. glabrata* *C. dubliniensis* *C. tropicalis* *C. Krusei*	Combinations or alone: Cyclosporine Sirolimus Tacrolimus Azathioprine Mycophenolate	Prevalence: Transplant group = 10.8% (*n* = 20) Control group: 1.4% (*n* = 1)	Pseudomembranous candidiasis: Transplant group = 3.2% (*n* = 6) Control group = 1.4% (*n* = 1) Atrophic Candidiasis: Transplant group = 7.6% (*n* = 14) Control group = 0% (*n* = 0)	3.5 (SD: 2.5) years	Not specified
López‐Pintor et al. (2010, 2013) [29, 30] Spain	Case‐control	Transplant group = 500 Control group = 501	Kidney	Transplant group = 53.63 (SD:13.42) Control group = 52.25 (SD:15.00)	Transplant group: Males = 307 Females = 193 Control group: Males = 314 Females = 187	Clinical examination, smear examination, culture examination and positive response to anti‐fungal treatment	Not specified	Azathioprine Cyclosporine Mycophenolate Prednisolone Sirolimus Tacrolimus	Prevalence: Transplant group = 7.4% (*n* = 37) Control group = 4.19% (*n* = 21)	Unclear distribution between the transplant group and control group	59.66 (SD: 55.81) months	Diabetes: Transplant group = 17% (*n* = 85) Control group = 3.2% (*n* = 16) Denture wearers: Transplant group = 27% (*n* = 135) Control group = 20% (*n* = 100) Active smoking: Transplant group = 20.6% (*n* = 103) Control group = 28.3% (*n* = 142) Smoking history: Transplant group = 24.4% (*n* = 122) Control group = 9.78% (*n* = 49) Alcoholics: Transplant group = 14.4% (*n* = 72) Control group = 24.2% (*n* = 121) Denture wearers: Transplant group = 27% (*n* = 35) Control group = 20% (*n* = 100)
Imko‐Walczuk et al. (2014) [31] Poland	Case‐control	Transplant group = 223 Control group = 100	Kidney	Transplant group = 48.77 (SD: 13.94) Control group = 45.5 (SD: 20.4)	Transplant group: Males = 126 Females = 97 Control group: Males = 61 Females = 39	Clinical examination with KOH staining, mucosal swabs and smear examination, culture on CHROMagar Candida medium and Sabouraud's medium	*C. albicans* *C. tropicana* *C. glabrata* *C. krusei* *C. famata*	Azathioprine Cyclosporine Mycophenolate Methyl prednisolone Prednisolone Tacrolimus	Prevalence: % in transplant group; % in control group?? Oral candidiasis C. albicans RTR = 74 patients (92%), Candida tropicana in 3 subjects (4%), other Candida species in 3 subjects (4%). Control = 21 (70%)	Pseudomembranous candidiasis Erythematous candidiasis	61 months (range:11 days‐300 months)	Not specified
Rezvani et al. (2014) [32] Iran	Cohort	Transplant = 59	Kidney	Mean age = 37 (range: 13–70)	Males = 41 Females = 18	Clinical examination, smear examination and microscopic examination	Not specified	Cyclosporine Mycophenolate Prednisolone	Incidence: 8.5% over 4 months Before transplant = 0% (*n* = 0) 4‐months post‐ transplant = 8.5% (*n* = 5)	Erythematous candidiasis (*n* = 5)	4 months	—
Zarei et al. (2020)^b^ [33] Iran	Prospective cohort study	Transplant = 81	Kidney (*n* = 40) Liver (*n* = 30) Heart (*n* = 10) Lung (*n* = 1) Bone marrow (*n* = 44)	Mean age = 52.2 (range: 15–75)	Males = 86 Females = 39	Clinical examination, culture on CHROMagar Candida medium and Sabouraud medium, Polymerase chain reaction‐restriction fragment length polymorphism (PCR‐RFLP)	*C. albicans* *C. glabrata* *C. parapsilosis* *C. tropicalis*	Not specified	Before transplant = 2,After transplant = 9] Bone marrow transplant group: before transplant = 3, after transplant = 6 Kidney transplant group: before = 1, after = 3 Liver transplant group: before = 1, after = 5 Heart transplant group: after = 1 None in Lung transplant group	Pseudomembranous candidiasis Erythematous candidiasis	6 months	Not specified
Ghadimi et al. (2023) [34] Iran	Prospective cohort study	Transplant = 40	Kidney	Range = 18 to 67 years	Males = 27 Females = 13	Clinical examination, culture on CHROMagar Candida medium and Sabouraud medium, and molecular identification using PCR	*C. albicans* *C. glabrata* *Rhodotorula*	CellCept R –methylprednisolone–Prograf R –anti‐thymocyte globulin	Before transplant = 20, After transplant = 25	Not specified	Not specified	Not specified

^a^ The total number of patients who received solid organ transplants was 125; however, data pertaining to 81 patients who received solid organ transplants was considered.

^b^ Only patients that had clinical symptoms with confirmed microbial diagnosis in the original articles were considered to have candidiasis.

### 3.3 Risk of Bias Within Studies

Table 2 shows the quality and bias assessment of the included case‐control and cohort studies using the NOS. For the case‐control studies, out of nine studies, within the selection domain, two studies received 2 stars [22, 28], five studies (equal to six articles) received 3 stars [24, 25, 27, 29, 30], while only two studies could be awarded 4 stars [23, 26] (which included randomized selected controls or community controls). In the comparability domain, only two studies (equal to three articles) [24, 29, 30] received a maximum of 2 stars, which considered diabetes, smoking, and removable prosthesis as the most important confounding factors, and age and gender as additional confounding factors, or balanced for these factors in data analysis, and one study [31] received 1 star.

**TABLE 2:** Quality assessment using the Newcastle Ottawa Scale [20].

Case‐control studies				
Author (Year)	Selection (Maximum 4 stars)	Comparability (Maximum 2 stars)	Exposure (Maximum 3 stars)	Total (Maximum 9 stars)
King et al. (1994) [22]	**	‒	**	****
Gülec et al. (2003) [23]	****	‒	**	******
Al‐Mohaya et al. (2009) [24]	***	**	**	*******
Antoniewicz et al. (2009) [25]	***	‒	**	*****
Dongari‐Bagtzoglou et al. (2009) [26]	****	‒	**	******
Gülec et al. (2010) [27]	***	‒	**	*****
Olczak‐Kowalczyk et al. (2010) [28]	**	‒	**	****
López‐Pintor et al. (2010, 2013) [29, 30]	***	**	**	*******
Imko‐Walczuk et al. (2014) [31]	***	*	**	*****

Cohort Studies				
Author (Year)	Selection (Maximum 4 stars)	Comparability (Maximum 2 stars)	Outcome (Maximum 3 stars)	Total (Maximum 9 stars)
Rezvani et al. (2014) [32]	****	‒	**	********
Zarei et al. (2020) [33]	***	‒	***	******
Ghadimi et al. (2023) [34]	***	‒	***	******

### 3.4 Synthesis of Results

**FIGURE 2:**
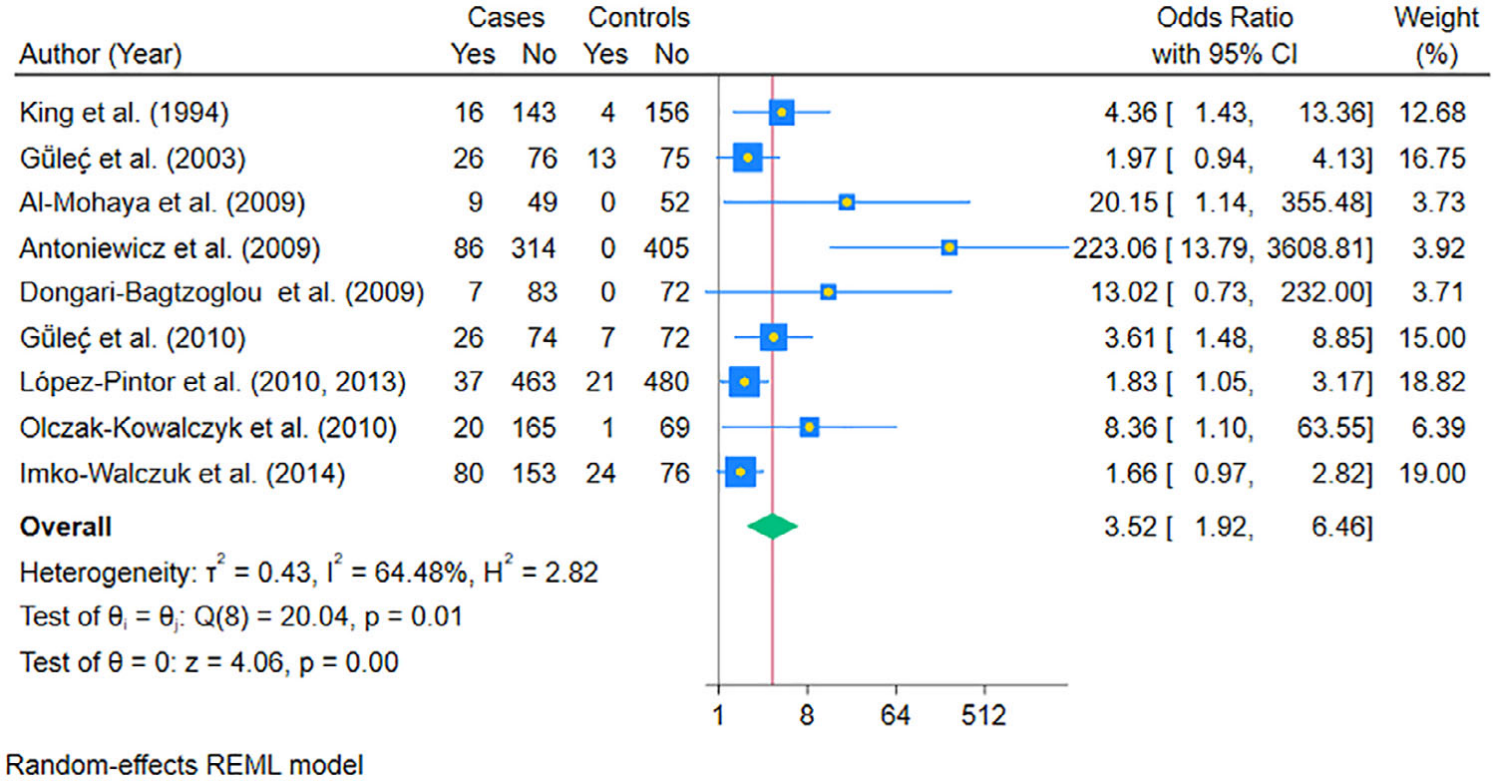
Forest plot of the pooled Odds Ratio (OR) of Oral Candidiasis in patients with solid organ transplants compared to the controls. The blue horizontal line for each study depicts the 95% confidence intervals (CI), the blue square represents the relative weighting, the yellow dot inside the blue square is the estimate of the study, and the green diamond is the 95% CI of the pooled estimate

Nine case‐control studies could be pooled to compare the Odds of candidiasis in cases compared to controls using a random‐effects REML model (Figure 2). The pooled OR of oral candidiasis in cases was found to be 3.52 (95% CI: 1.92, 6.46; *p* < 0.05) compared to controls. The statistical heterogeneity was high, as indicated by the I^2^ value of 64.48% (*p* < 0.05).

### 3.5 Sensitivity Analysis

**FIGURE 3:**
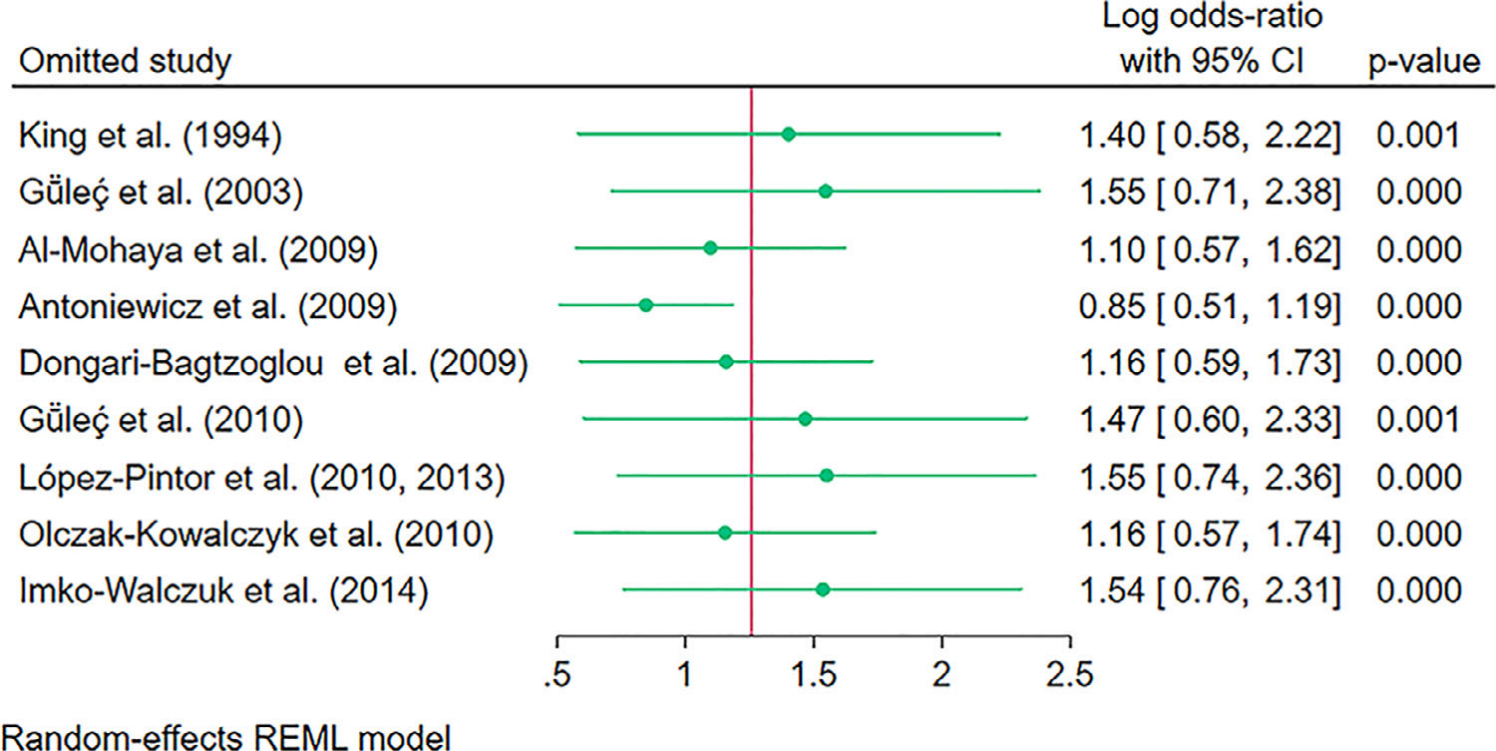
Sensitivity analysis using *“leave‐one‐out”* approach for the pooled Odds Ratio (OR) of Oral Candidiasis in patients with solid organ transplants compared to the controls. The green horizontal line with the green dot depicts the 95% confidence intervals (CI) and the pooled Odds Ratio (OR) if that study is excluded from the analysis

To identify if any particular study had a high effect on the pooled estimate, a sensitivity analysis was conducted using a *leave‐one‐out* approach (Figure 3). The results indicated that the results remained statistically significant if any single study was omitted from the pooled analysis, and the model was robust.

### 3.6 Publication Bias and Small Study Effects

**FIGURE 4:**
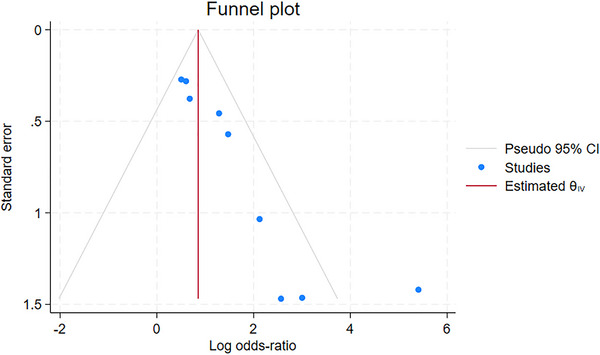
Funnel plot to assess the publication bias

Publication bias was assessed by generating a funnel plot. As seen in Figure 4, the funnel plot was asymmetrical, indicating publication bias. To verify the results, we conducted a regression‐based Egger test for small study effects, which was found to be statistically significant (*z* = 4.10; *p* < 0.05). The results of the funnel plot and regression‐based Egger test might indicate that smaller studies tended to have larger effect sizes and thus might have publication bias.

### 3.7 Subgroup Analysis

**FIGURE 5:**
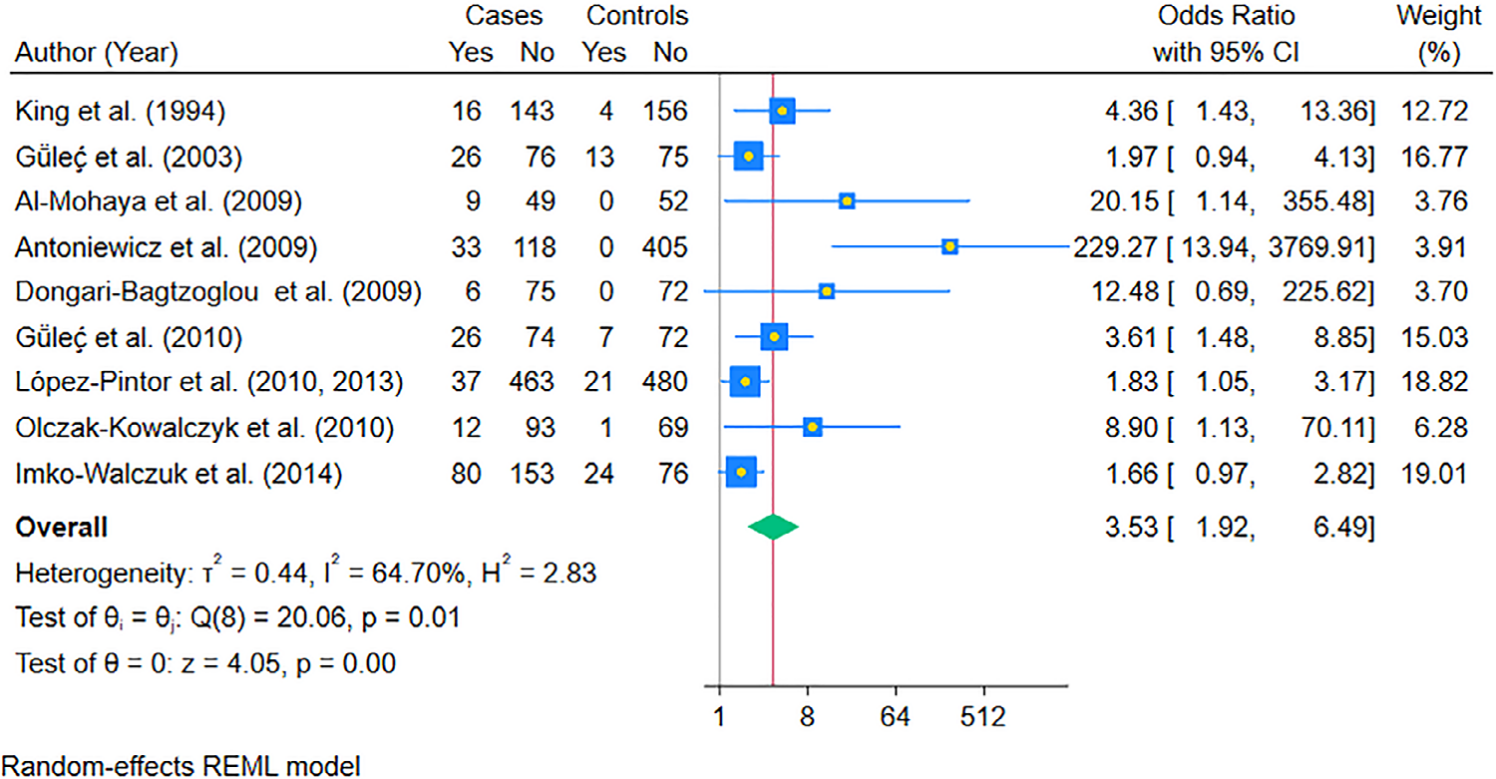
Forest plot of the pooled Odds Ratio (OR) of Oral Candidiasis in patients with Kidney transplants compared to the controls. The blue horizontal line for each study depicts the 95% confidence intervals (CI), the blue square represents the relative weighting, the yellow dot inside the blue square is the estimate of the study, and the green diamond is the 95% CI

**FIGURE 6:**
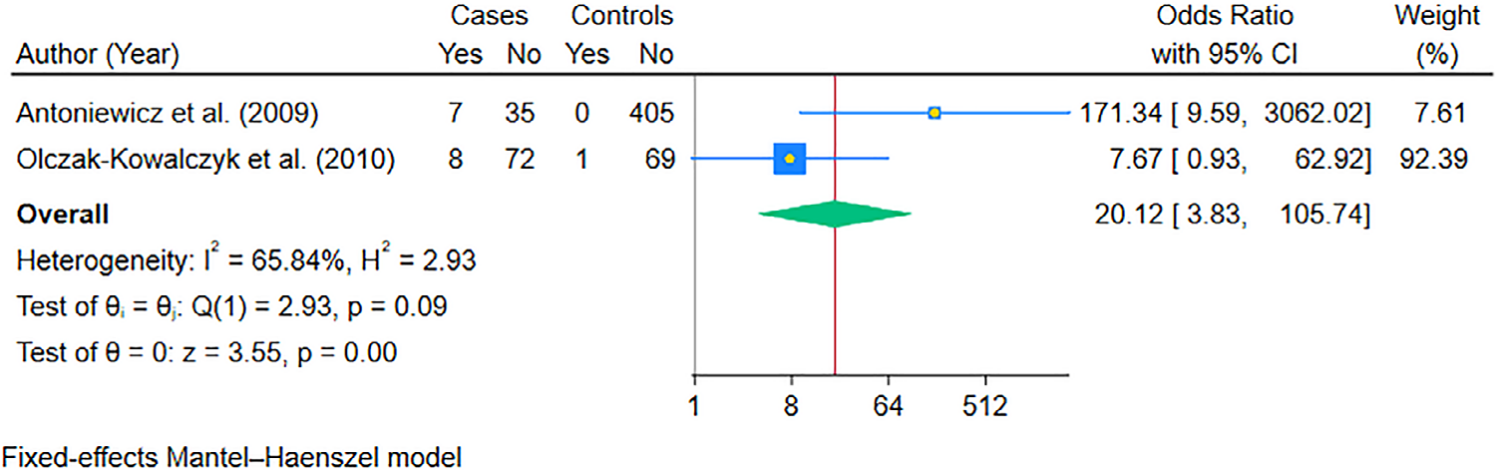
Forest plot of the pooled Odds Ratio (OR) of Oral Candidiasis in patients with Liver transplants compared to the controls. The blue horizontal line for each study depicts the 95% confidence intervals (CI), the blue square represents the relative weighting, the yellow dot inside the blue square is the estimate of the study, and the green diamond is the 95% CI

**FIGURE 7:**
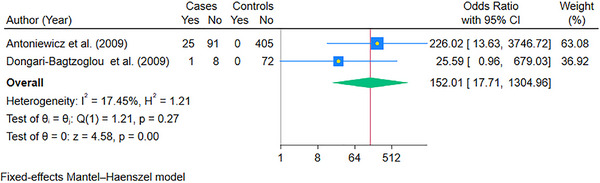
Forest plot of the pooled Odds Ratio (OR) of Oral Candidiasis in patients with Heart transplants compared to the controls. The blue horizontal line for each study depicts the 95% confidence intervals (CI), the blue square represents the relative weighting, the yellow dot inside the blue square is the estimate of the study, and the green diamond is the 95% CI

To account for the high statistical heterogeneity, we performed subgroup analysis according to the solid organ transplanted. The pooled OR of oral candidiasis in kidney transplant recipients (9 studies) was 3.53 (95% CI: 1.92, 6.49; *p* < 0.05) (Figure 5). The studies that could be pooled for liver (Figure 6) and heart (Figure 7) were minimal, with pooled ORs of 20.12 (95% CI: 3.83, 105.74; *p* < 0.05) and 152.01 (95% CI: 17.71, 1304.96; *p* < 0.05), respectively. The high values of ORs were primarily due to no oral candidiasis in the controls. Finally, the results for lung transplant recipients could not be pooled due to a single case‐control study that could be included [25]; however, the calculated OR was 247.33 (95% CI: 14.81, 4129.63), which was high due to the same reason as mentioned above.

### 3.8 Certainty of Assessment

There was moderate certainty of evidence that there were higher odds of oral candidiasis in immunosuppressed patients who received solid organ transplants compared to controls (Table 3). Similarly, the evidence was moderate for subgroup analysis regarding the kidney transplant recipients, liver transplant recipients, and heart transplant recipients.

**TABLE 3:** GRADE [21] evidence profile (using GRADEpro GDT).

												Summary of findings		
	Certainty assessment							Number of cases		Number of controls		Effect		
Risk of oral candidiasis	Number of studies	Study design	Risk of bias	Inconsistency^a^	Indirectness	Imprecision^b^	Other considerations^c^	Events (*N*)	No events (*N*)	Events (*N*)	No events (*N*)	Relative (95% CI)	Absolute (95% CI)	Certainty of Evidence
Solid organ transplants														
	9	Case‐control	Not serious	Not serious	Not serious	Not serious	Strong association	307	1520	70	1457	OR 3.52 (1.92, 6.46)	112 more per 1000 (from 44 more to 213 more)	⨁⨁⨁◯ Moderate
Kidney transplant														
	9	Case‐control	Not serious	Not serious	Not serious	Not serious	Strong association	245	1244	70	1457	OR 3.53 (1.92, 6.49)	112 more per 1000 (from 44 more to 214 more)	⨁⨁⨁◯ Moderate
Lung transplant														
	1	Case‐control	Not serious	Not serious	Not serious	Serious	Very strong association	21	70	0	405	OR 247.33 (14.81, 4129.63)	880 more per 1000 (from 401 more to 942 more)	⨁⨁⨁◯ Moderate
Liver transplant														
	2	Case‐control	Not serious	Not serious	Not serious	Serious	Very strong association	15	107	1	474	OR 20.12 (3.83, 105.74)	478 more per 1000 (from 124 more to 803 more)	⨁⨁⨁◯ Moderate
Heart transplant														
	2	Case‐control	Not serious	Not serious	Not serious	Serious	Very strong association	26	99	0	477	OR 89.91 (10.66, 758.60)	842 more per 1000 (from 446 fewer to 933 more)	⨁⨁⨁◯ Moderate

^a^ Inconsistency: Downgraded if the effect size was in the opposite direction after conducting a sensitivity analysis.

^b^ Imprecision: Downgraded as the OIS (optimal information size) criteria were not met: 300 events in the case of dichotomous outcomes. Moreover, the confidence intervals did not overlap [33].

^c^ OR of 3–6 was considered a strong association, whereas OR above 6 was considered a very strong association between the exposure and the event, and the level of evidence was upgraded accordingly. OR less than 3 was not considered strong enough and thus was not upgraded.

## 4 Discussion

Even though oral candidiasis is not fatal, and its symptoms might remain occult in patients’ oral cavities, the disease requires urgent treatment, especially in immunocompromised individuals. This is due to its propensity to disseminate systemically to other sites like the pharynx, lungs, or esophagus, and eventually might become life‐threatening. Furthermore, invasive candidal infection and superficial candidal infection have been shown to pose a huge economic burden in terms of direct medical costs for hospitalizations and the number of outpatient visits [35, 36]. The present systematic review and meta‐analyses were thus done to evaluate the association of oral candidiasis in patients who received solid organ transplants. There were almost three and a half times higher odds of developing candidiasis in patients who received solid organ transplants compared to the healthy controls. The odds of oral candidiasis in patients who received kidney transplants were almost 3.53 times higher, whereas those who received liver transplants had almost 20 times higher odds. The same has also been reported by other authors that liver transplant recipients have a higher rate of Candida infections compared to other organs [37]. Higher infections in liver transplant recipients might be related to the complex surgery and prolonged surgery time [38]. Another reason for the lower OR of oral candidiasis in patients with kidney transplants (OR: 3.52) compared to patients with liver transplants (OR: 20.12) might be attributed to higher survival and growth of Candida albicans in an acidic environment, whereas in chronic kidney patients, the pH is less acidic (or alkaline) [39, 40]. It is prudent to mention that the results of meta‐analysis for the risk of oral candidiasis in patients with lung and heart transplants should be interpreted with caution due to the limited number of studies and data from a limited number of patients that could be pooled. Some of the included studies had no patients with oral candidiasis in the control group [24, 25, 26]; thus, calculating OR using the original data from the studies would have theoretically given an infinite value (due to a zero in a cell in the 2 by 2 table). In analysis involving sparse data with zero outcome counts in one cell, the statistical software applied a continuity correction [41] (adding 0.5 to all cells) to compute the odds ratio. Although this approach enabled the calculation of a finite odds ratio, it is an arbitrary adjustment that can bias effect estimates toward the null and inflate variances, particularly in settings with rare events and imbalanced group sizes. This correction may have influenced the magnitude & precision of the estimated association, and thus the pooled OR with the above studies should be interpreted cautiously and in conjunction with the results of the sensitivity analysis utilizing the “*leave‐one‐out*” approach by excluding those particular studies. No study could be found that evaluated the risk of oral candidiasis in patients with other solid organ transplants (pancreas, small bowel). Also, it is noteworthy to mention that since most of the included studies were case‐control studies, we could establish an association but not a causation. Future long‐term cohort studies with adequate follow‐up could establish the temporality and infer causality.

It is essential to understand the subtle distinction between oral candidal colonization and oral candidal infection (oral candidiasis) to provide relevance and accuracy of results in the context of the study, because the primary aim of the study was to assess the risk of oral candidiasis (and not oral colonization) in organ transplant recipients. Since Candida is a normal commensal of the oral cavity, oral candidal colonization is pertinent in both transplant recipients and healthy controls. Hence, standardization/clarity of the terms was achieved by the following definition: Positive cultures without clinical symptoms and oral lesions were considered to be candidal colonization, while positive direct examination and culture concurrent with symptoms such as erythema, pseudomembrane in oral mucosa, dry mouth, and glossalgia were regarded as oral candidiasis. The diagnosis of oral candidiasis was based on the presence of clinical symptoms (burning), with white scrapable plaque (pseudomembrane) in case of pseudomembranous candidiasis and erythematous areas in erythematous candidiasis and substantiated (confirmed) by demonstration of candidal hyphae in cytological smears, positive culture in Sabouraud dextrose agar, and histopathological evaluation of biopsied specimens, if necessary, or by advanced diagnostic methods like polymerase chain reaction and germ tube test. Thus, many newer studies (Appendix S2) were excluded that evaluated Oral Candidiasis based only on clinical criteria, which might not be appropriate to confirm Candidiasis.

When there is an imbalance under certain conditions like diabetes mellitus, anemia, nutritional deficiency, prolonged hospitalizations, or predisposing factors like old age, dentures, heavy smoking, and prolonged use of antibiotics and corticosteroids, normal commensal may transform into a pathogenic form, but the mere presence of the fungus (oral colonization) does not indicate disease and requires actual penetration of the tissues to be considered as infection (oral candidiasis). Hence, in our review, we have only considered diabetes mellitus, smoking, dentures, or other immunocompromised conditions as the most important confounding factors, and age as the second most important confounding factor, as they directly affect the prognosis of patients with oral candidiasis and patients undergoing solid organ transplant.

A thorough systematic search, robust meta‐analyses including sensitivity analysis, assessment of the statistical heterogeneity, subgroup analysis, and the use of the GRADE approach to assess the certainty of evidence are some of the key strengths of this systematic review. However, high heterogeneity was one of the limitations of the review, which is not related to the methodology of the review. We tried to identify the sources of statistical heterogeneity by conducting subgroup analysis and sensitivity analysis. The different regimes of immunosuppressants, underlying medical disorders (diabetes), different post‐transplantation periods, and other unknown factors affecting the immunity of an individual might be able to account for the high statistical heterogeneity. We also intended to conduct a subgroup analysis based on the immunosuppressant regimen as per our protocol; however, due to the heterogeneity in different regimes and studies, this was not possible. Also, some of the studies included in the review are older, and the different immunosuppressant regimes might have impacted the results. Nevertheless, this systematic review and meta‐analyses add significantly to our understanding of oral candidiasis in patients with solid organ transplants, and these limitations are not inherent to the methodology per se, but rather due to the quality of the studies that could be included due to our strict inclusion criteria. Since there were only a few studies that evaluated the association of oral candidiasis in liver, lung, and heart transplant recipients, and no study that evaluated oral candidiasis in small‐bowel or pancreas recipients, more studies in these patients would be the way forward to add to the science of infectious disease in organ transplantation. Consequently, future well‐designed studies using robust and standardized methodologies, with better control of the confounding factors that might be the risk factors for oral candidiasis, are important, given the increased risk of oropharyngeal candidiasis observed in immunosuppressed transplant recipients as concluded in our review, future research should also focus on evaluating the effectiveness and optimal use of antifungal prophylaxis as a potential strategy to mitigate this risk [42, 43].

## 5 Conclusions

There was a moderate certainty of evidence that immunosuppressed individuals receiving solid organ transplants had a higher risk of oral candidiasis compared to healthy controls. Also, there was a moderate certainty of evidence that immunosuppressed individuals receiving kidney, liver, lung, or heart transplants had a higher risk of oral candidiasis. There were no studies that evaluated the association of oral candidiasis in patients who received pancreas or small bowel transplants.

## Author Contributions

S.N., S.S., S.A., and S.D. conceptualized & designed the study, coordinated data collection, and drafted the manuscript. S.D. conducted the analyss and supervised the project. All the authors gave final approval of the manuscript before submission and agreed to be accountable for all aspects of the work, ensuring integrity and accuracy.

## Funding

The authors have nothing to report

## Conflicts of Interest

The authors declare no conflicts of interest.

## Statement of Institutional Review Board Approval or Waiver

The study did not involve any human participants or research on animals. Thus, the institutional review board approval was not required or sought.

## Registration

PROSPERO (International Prospective Register of Systematic Reviews) registration number. The protocol of the review is freely available from: https://www.crd.york.ac.uk/prospero/display_record.php?RecordID=363816.

## Supporting information




**Appendix S1**: Search Strategy (March 7, 2025)

**Appendix S2**: Reasons for exclusion
