# Supplementary material for: Association Between Solid Organ Transplantation and Oral Candidiasis: A Systematic Review and Meta‐Analysis
Source: Spec Care Dentist. 2026 Mar 23;46(2):e70157. doi: 10.1111/scd.70157 (PMC13007491; doi:10.1111/scd.70157)
Supplement: Supplementary file 1 — Appendix S1: Search Strategy (March 7, 2025) [file SCD-46-0-s002.docx]

**Appendix 1: Search Strategy (March 7, 2025)**

**PubMed: 251 results**

**("candida"[MeSH Terms] OR "candida"[All Fields] OR "candidae"[All Fields] OR "candidas"[All Fields] OR ("candidiasis"[MeSH Terms] OR "candidiasis"[All Fields] OR "candidiases"[All Fields]) OR ("candidiasis, oral"[MeSH Terms] OR ("candidiasis"[All Fields] AND "oral"[All Fields]) OR "oral candidiasis"[All Fields] OR "thrush"[All Fields] OR "candidiasis"[MeSH Terms] OR "candidiasis"[All Fields] OR "thrushes"[All Fields]) OR ("candidiasis"[MeSH Terms] OR "candidiasis"[All Fields] OR "moniliasis"[All Fields])) AND ("mouth"[MeSH Terms] OR "mouth"[All Fields] OR "oral"[All Fields] OR ("teeth s"[All Fields] OR "teeths"[All Fields] OR "tooth"[MeSH Terms] OR "tooth"[All Fields] OR "teeth"[All Fields] OR "tooth s"[All Fields] OR "tooths"[All Fields]) OR ("mouth"[MeSH Terms] OR "mouth"[All Fields] OR "mouths"[All Fields] OR "mouth s"[All Fields] OR "mouthed"[All Fields] OR "mouthful"[All Fields] OR "mouthfuls"[All Fields] OR "mouthing"[All Fields]) OR ("tongue"[MeSH Terms] OR "tongue"[All Fields] OR "tongues"[All Fields] OR "tongue s"[All Fields]) OR ("buccal"[All Fields] OR "buccally"[All Fields])) AND ((("solid"[All Fields] OR "solid s"[All Fields] OR "solids"[All Fields]) AND ("transplants"[MeSH Terms] OR "transplants"[All Fields] OR ("organ"[All Fields] AND "transplant"[All Fields]) OR "organ transplant"[All Fields] OR "organ transplantation"[MeSH Terms] OR ("organ"[All Fields] AND "transplantation"[All Fields]) OR "organ transplantation"[All Fields] OR ("organ"[All Fields] AND "transplant"[All Fields]))) OR ("kidney transplantation"[MeSH Terms] OR ("kidney"[All Fields] AND "transplantation"[All Fields]) OR "kidney transplantation"[All Fields] OR ("kidney"[All Fields] AND "transplant"[All Fields]) OR "kidney transplant"[All Fields]) OR ("heart transplantation"[MeSH Terms] OR ("heart"[All Fields] AND "transplantation"[All Fields]) OR "heart transplantation"[All Fields] OR ("heart"[All Fields] AND "transplant"[All Fields]) OR "heart transplant"[All Fields]) OR ("liver transplantation"[MeSH Terms] OR ("liver"[All Fields] AND "transplantation"[All Fields]) OR "liver transplantation"[All Fields] OR ("liver"[All Fields] AND "transplant"[All Fields]) OR "liver transplant"[All Fields]) OR ("lung transplantation"[MeSH Terms] OR ("lung"[All Fields] AND "transplantation"[All Fields]) OR "lung transplantation"[All Fields] OR ("lung"[All Fields] AND "transplant"[All Fields]) OR "lung transplant"[All Fields]) OR (("intestinalization"[All Fields] OR "intestinalized"[All Fields] OR "intestinally"[All Fields] OR "intestinals"[All Fields] OR "intestine s"[All Fields] OR "intestines"[MeSH Terms] OR "intestines"[All Fields] OR "intestinal"[All Fields] OR "intestine"[All Fields]) AND ("transplantability"[All Fields] OR "transplantable"[All Fields] OR "transplantated"[All Fields] OR "transplantating"[All Fields] OR "transplantation"[MeSH Terms] OR "transplantation"[All Fields] OR "transplantations"[All Fields] OR "transplanted"[All Fields] OR "transplanting"[All Fields] OR "transplantation"[MeSH Subheading] OR "transplantation s"[All Fields] OR "transplanter"[All Fields] OR "transplanters"[All Fields] OR "transplantion"[All Fields] OR "transplants"[MeSH Terms] OR "transplants"[All Fields] OR "transplant"[All Fields])) OR ("pancreas transplantation"[MeSH Terms] OR ("pancreas"[All Fields] AND "transplantation"[All Fields]) OR "pancreas transplantation"[All Fields] OR ("pancreas"[All Fields] AND "transplant"[All Fields]) OR "pancreas transplant"[All Fields]))**

**Medline: 117 results**

Ovid MEDLINE(R) ALL 1946 to March 06, 2025

| S No. | Keywords | Number of yields |
| --- | --- | --- |
|  | oral candidiasis.mp. or exp Candidiasis, Oral/ | 6499 |
|  | exp Candida/ or exp Candida albicans/ or oral candida.mp. | 53728 |
|  | Thrush.mp | 1546 |
|  | moniliasis.mp. | 2068 |
|  | 1 or 2 or 3 or 4 | 60542 |
|  | oral.mp. or exp Oral Health/ or exp Pathology, Oral/ | 869579 |
|  | exp Mouth Diseases/ or exp Mouth Floor/ or exp Mouth/ or exp Mouth Mucosa/ or mouth.mp. | 660841 |
|  | 6 or 7 | 1378598 |
|  | exp Organ Transplantation/ or exp Kidney Transplantation/ or solid organ transplant.mp. or exp Transplant Recipients/ | 250550 |
|  | exp Heart Transplantation/ or heart transplant.mp. | 44415 |
|  | liver transplant.mp. or exp Liver Transplantation/ | 73972 |
|  | exp Kidney Transplantation/ or kidney transplant.mp. | 116523 |
|  | intestine transplant.mp. | 146 |
|  | exp Lung Transplantation/ or lung transplant.mp. | 22562 |
|  | exp Pancreas Transplantation/ or pancreas transplant.mp. | 8305 |
|  | 9 or 10 or 11 or 12 or 13 or 14 or 15 | 267858 |
|  | 5 and 8 and 16 | 117 |

**Embase: 776**

Embase 1974 to 2025 March 06

| S No. | Keywords | Number of yields |
| --- | --- | --- |
|  | oral candidiasis.mp. or exp Candidiasis, Oral/ | 11646 |
|  | exp Candida/ or exp Candida albicans/ or oral candida.mp. | 108173 |
|  | Thrush.mp | 11809 |
|  | moniliasis.mp. | 272 |
|  | 1 or 2 or 3 or 4 | 118192 |
|  | oral.mp. or exp Oral Health/ or exp Pathology, Oral/ | 3040317 |
|  | exp Mouth Diseases/ or exp Mouth Floor/ or exp Mouth/ or exp Mouth Mucosa/ or mouth.mp. | 961161 |
|  | 6 or 7 | 3659922 |
|  | exp Organ Transplantation/ or exp Kidney Transplantation/ or solid organ transplant.mp. or exp Transplant Recipients/ | 518915 |
|  | exp Heart Transplantation/ or heart transplant.mp. | 86196 |
|  | liver transplant.mp. or exp Liver Transplantation/ | 155128 |
|  | exp Kidney Transplantation/ or kidney transplant.mp. | 199494 |
|  | intestine transplant.mp. | 264 |
|  | exp Lung Transplantation/ or lung transplant.mp. | 52249 |
|  | exp Pancreas Transplantation/ or pancreas transplant.mp. | 23474 |
|  | 9 or 10 or 11 or 12 or 13 or 14 or 15 | 529953 |
|  | 5 and 8 and 16 | 776 |

**Scopus: 370**

( TITLE-ABS-KEY ( ( candidiasis ) OR ( candida ) OR ( thrush ) OR ( moniliasis ) ) AND TITLE-ABS-KEY ( ( oral ) OR ( mouth ) ) AND TITLE-ABS-KEY ( ( ( solid AND organ AND transplant ) OR ( kidney AND transplant ) OR ( liver AND transplant ) OR ( lung AND transplant ) OR ( intestine AND transplant ) OR ( pancreas AND transplant ) OR ( heart AND transplant ) ) ) )

**Web of Science: 169**

((ALL=((candidiasis OR candida OR thrush OR moniliasis) )) AND ALL=((oral OR mouth OR teeth) )) AND ALL=(((solid organ transplant) OR (heart transplant) OR (lung transplant) OR (liver transplant) OR (intestine transplant) OR (kidney transplant) OR (pancreas transplant)))
