# Supplementary material for: Association Between Solid Organ Transplantation and Oral Candidiasis: A Systematic Review and Meta‐Analysis
Source: Spec Care Dentist. 2026 Mar 23;46(2):e70157. doi: 10.1111/scd.70157 (PMC13007491; doi:10.1111/scd.70157)
Supplement: Supplementary file 2 — Appendix S2: Reasons for exclusion [file SCD-46-0-s001.docx]

**Appendix 2: Reasons for exclusion**

| **S. No.** | **Study** | **Reason** |
| --- | --- | --- |
|  | Helenius-Hietala J, Ruokonen H, Grönroos L, Rissanen H, Vehkalahti MM, Suominen L, Isoniemi H, Meurman JH. Oral mucosal lesions in liver transplant recipients and controls. Transplant International. 2013;2:138 | Conference Abstract |
|  | Olczak-Kowalczyk D, Pawlowska J, Garczewska B, Smirska E, Syczewska M, Grenda R.Oral Candida infection in organ transplanted patients. Pediatric Transplantation. 2009;1:64 | Conference Abstract |
|  | Lopez-Pintor RM, Hernandez G, De Arriba L, Alcaide A, De Andre s A. Comparison of oral candidiasis prevalence in renal transplants and controls. Oral Dis. 2010;16:531 | Conference Abstract |
|  | Dirschnabel AJ, Martins Ade S, Dantas SA, Ribas Mde O, Grégio AM, Alanis LR, Ignacio SA, Trevilatto PC, Casagrande RW, de Lima AA, Machado MÂ. Clinical oral findings in dialysis and kidney-transplant patients. Quintessence Int. 2011;42:127 | Only clinical diagnosis |
|  | Kaswan S, Patil S, Maheshwari S, Wadhawan R. Prevalence of oral lesions in kidney transplant patients: A single center experience. Saudi J Kidney Dis Transpl. 2015;26:678 | Only clinical diagnosis |
|  | Sarmento DJS, Aires Antunes RSCC, Cristelli M, Braz-Silva PH, Maciel R, Pestana JOMA, Gallottini M. Oral manifestations of allograft recipients immediately before and after kidney transplantation. Acta Odontol Scand. 2020;78:217 | Only clinical diagnosis |
|  | Schander K, Jontell M, Johansson P, Nordén G, Hakeberg M, Bratel J. Oral infections and their influence on medical rehabilitation in kidney transplant patients. Swed Dent J. 2009;33:97 | Only clinical diagnosis |
|  | Helenius-Hietala J, Ruokonen H, Grönroos L, Rissanen H, Vehkalahti MM, Suominen L, Isoniemi H, Meurman JH. Oral mucosal health in liver transplant recipients and controls. Liver Transpl. 2014;20:72 | Only clinical diagnosis |
|  | Pasternak Y, Rubin S, Bilavsky E, Mozer-Glassberg Y, Levy I, Nahum E, Rom E, Gurevich M, Ben-Zvi H, Ashkenazi-Hoffnung L. Risk factors for early invasive fungal infections in paediatric liver transplant recipients. Mycoses. 2018;61:639 | Wrong comparator |
|  | Kurnatowska I, Chrzanowski W, Kacprzyk F, Kurnatowska A. Wieloogniskowe zarazenia grzybami u poddawanych immunosupresji osób po przeszczepach narek [Multifocal fungal infections in patients after renal transplantation undergoing immunosuppression]. Pol Merkur Lekarski. 2003;15:388 | Wrong comparator |
|  | De la Rosa-García E, Mondragón-Padilla A. Lesiones bucales asociadas a inmunosupresión en pacientes con trasplante renal [Oral lesions associated to immunosuppression in kidney transplant patients]. Rev Med Inst Mex Seguro Soc. 2014;52:442 | Wrong comparator |
|  | Olivas-Escárcega V, Rui-Rodríguez Mdel S, Fonseca-Leal Mdel P, Santos-Díaz MA, Gordillo-Moscoso A, Nernández-Sierra JF, de J Pozos-Guillén A. Prevalence of oral candidiasis in chronic renal failure and renal transplant pediatric patients. J Clin Pediatr Dent. 2008;32:313 | Wrong comparator (control population is not healthy control) |
|  | Sabadin CES, Lopes SL, Gompertz OF, Santana GNP, de Azevedo Melo AS, Rigo L, Da Matta DA, Barbosa DA. Oral colonization by Candida spp. in liver transplant patients: Molecular identification and antifungal susceptibility: Oral colonization by Candida spp. in liver transplant. Med Mycol. 202;59:578 | Wrong outcomes |
|  | Caliento R, Sarmento DJS, Silva ÉMP, Tozetto-Mendoza TR, Tobouti PL, Benini V, Braz-Silva PH, Gallottini M. Oral shedding of HSV-1 and EBV and oral manifestations in paediatric chronic kidney disease patients and renal transplant recipients. Acta Odontol Scand. 2018;76:539 | Wrong outcomes |
|  | Khedr L, Teama N, El Sharkawy M. Infections in the first year of living related kidney transplantation in a young transplant cohort. BMC Nephrol. 2023;24:328 | Wrong outcomes |
|  | Ribeiro PM, Bacal F, Koga-Ito CY, Junqueira JC, Jorge AO. Presence of Candida spp. in the oral cavity of heart transplantation patients. J Appl Oral Sci. 2011;19:6 | Wrong outcomes |
|  | Pindycka-Piaszczynska M, Gebska E, Wojarski J, Bendkowski W, Zembala M. Oral candidiasis in heart transplant recipients. Pol J Immunol. 1995;20:109 | Wrong outcomes |
|  | Ju C, Lian Q, Xu X, Cao Q, Lan C, Chen R, He J. Epidemiology and Prognosis of Invasive Fungal Disease in Chinese Lung Transplant Recipients. Front Med (Lausanne). 2021;8:718747 | Wrong outcomes |
|  | Al Nowaiser A, Lucas VS, Wilson M, Roberts GJ, Trompeter RS. Oral health and caries related microflora in children during the first three months following renal transplantation. Int J Paediatr Dent. 2004;14:118 | Wrong outcomes |
|  | Sganga G, Bianco G, Frongillo F, Lirosi MC, Nure E, Agnes S. Fungal infections after liver transplantation: incidence and outcome. Transplant Proc. 2014;46:2314 | Wrong outcomes |
|  | Nylund KM, Meurman JH, Heikkinen AM, Furuholm JO, Ortiz F, Ruokonen HM. Oral health in patients with renal disease: a longitudinal study from predialysis to kidney transplantation. Clin Oral Investig. 2018;22:339 | Wrong outcomes |
|  | Siahi-Benlarbi R, Nies SM, Sziegoleit A, Bauer J, Schranz D, Wetzel WE. Caries-, Candida- and Candida antigen/antibody frequency in children after heart transplantation and children with congenital heart disease. Pediatr Transplant. 2010;14:715 | Wrong outcomes |
|  | Sabadin CES, Matta DAD, Hoppe L, Fernandes FAV, Melo ASA, Rigo L, Barbosa DA. Oral candidiasis in liver transplant patients: species identification and antifungal susceptibility profile. Einstein (Sao Paulo). 2024;22:eAO0138. | Wrong outcomes |
|  | Olczak-Kowalczyk D, Pawłowska J, Cukrowska B, Kluge P, Witkowska-Vogtt E, Dzierzanowska-Fangrat K, Wrześniewska D, Smirska E, Grenda R. Local presence of cytomegalovirus and Candida species vs oral lesions in liver and kidney transplant recipients. Ann Transplant. 2008;13:28 | Wrong outcomes |
|  | Pappas PG, Andes D, Schuster M, Hadley S, Rabkin J, Merion RM, Kauffman CA, Huckabee C, Cloud GA, Dismukes WE, Karchmer AW. Invasive fungal infections in low-risk liver transplant recipients: a multi-center prospective observational study. Am J Transplant. 2006;6:386 | Wrong outcomes |
|  | Marinelli T, Pennington KM, Hamandi B, Donahoe L, Rotstein C, Martinu T, Husain S. Epidemiology of candidemia in lung transplant recipients and risk factors for candidemia in the early posttransplant period in the absence of universal antifungal prophylaxis. Transpl Infect Dis. 2022;24:e13812. | Wrong outcomes |
|  | Uip DE, Amato Neto V, Varejão Strabelli TM, Alcides Bocchi E, Fiorelli A, Stolf N, Bellotti G, Pileggi F, Jatene AD. Infecções fúngicas em 100 pacientes submetidos a transplante cardíaco [Fungal infections in 100 patients subjected to heart transplantation]. Arq Bras Cardiol. 1996;66:65 | Wrong study design |
|  | Sahebjamee M, Shakur Shahabi M, Nikoobakht MR, Momen Beitollahi J, Mansourian A. Oral lesions in kidney transplant patients. Iran J Kidney Dis. 2010;4:232 | Wrong study design |
|  | Yazdanpanah S, Shafiekhani M, Zare Z, Nikoupour H, Geramizadeh B, Chamanpara P, Jabrodini A, Ahmadi M, Malekizadeh Z, Anbardar MH, Pakshir K, Zomorodian K. Species distribution and antifungal susceptibility patterns of Candida involvement in pediatric solid organ transplant recipients: A cross-sectional study from a single transplant center. J Mycol Med. 2025;35:101522 | Wrong study design |
|  | Rabkin JM, Oroloff SL, Corless CL, Benner KG, Flora KD, Rosen HR, Olyaei AJ. Association of fungal infection and increased mortality in liver transplant recipients. Am J Surg. 2000;179:426 | Wrong study design |
|  | da Silva LC, de Almeida Freitas R, de Andrade MP Jr, Piva MR, Martins-Filho PR, de Santana Santos T. Oral lesions in renal transplant. J Craniofac Surg. 2012;23:e214 | Wrong study design |
|  | Pereira-Lopes O, Sampaio S, Vieira-Marques P, Monteiro-Da-Silva F, Felino A, Pestana M, Sampaio-Maia B. Oral candida prevalence in renal transplant recipients receiving cyclosporine, everolimus or tacrolimus and living kidney donors. Transplant International November 2013;2:199 | Wrong study design |
|  | Ko KS, Cho DO, Ahn JH, Lee TW, Ihm CG, Chang SG, Chai SE, Park HC, Hong SH, Joo HZ, et al. Infections after renal transplantation. Transplant Proc. 1994;26:2072-4. | Wrong study design |
|  | Spolidorio LC, Spolidorio DM, Massucato EM, Neppelenbroek KH, Campanha NH, Sanches MH. Oral health in renal transplant recipients administered cyclosporin A or tacrolimus. Oral Dis. 2006;12:309 | Wrong study design |
|  | Sammons C, Norris, M, Casciello, N. Evaluation of Oral Candidiasis Incidence at Large Academic Medical Center in Absence of Pharmacologic Prophylaxis at Discharge. American Journal of Transplantation. 2020;20(Supplement 3):797-798 | Wrong study design |
|  | Pappas PG, Silveira FP. Candida in solid organ transplant recipients. American Journal of Transplantation. 2009;9(SUPPL. 4):S173 | Wrong study design |
|  | Santos SBD, Sabadin CES, Mario DN, Rigo L, Barbosa DA. Presence of Candida spp. and candidiasis in liver transplant patients. An Bras Dermatol. 2018;93:356 | Wrong study design |
|  | Sánchez-Lázaro IJ, Almenar L, Blanes M, Martínez-Dolz L, Portolés M, Roselló E, Rivera M, Salvador A. Timing, etiology, and location of first infection in first year after heart transplantation. Transplant Proc. 2010;42:3017 | Wrong study design |
